# Multi-neuromeric origin of tyrosine hydroxylase-positive neurons within the substantia nigra and ventral tegmental area

**DOI:** 10.3389/fnana.2025.1612529

**Published:** 2025-05-30

**Authors:** José L. Ferran, Franco Lucero-Arteaga, Abdelmalik Ayad, Yevheniy Kutsenko, A. Alonso, B. Ribeiro Do-Couto, M. Á. García-Cabezas, Kuei Y. Tseng

**Affiliations:** ^1^Department of Human Anatomy and Psychobiology, School of Medicine, University of Murcia, Murcia, Spain; ^2^Institute of Biomedical Research of Murcia – IMIB, Virgen de la Arrixaca University Hospital, Murcia, Spain; ^3^Consejo Nacional de Investigaciones Científicas y Técnicas (CONICET), Buenos Aires, Argentina; ^4^Centro de Producción de Animales de Experimentación, Facultad de Ciencias Veterinarias, Universidad Nacional de La Pampa, General Pico, Argentina; ^5^Department of Human Anatomy and Psychobiology, Faculty of Psychology, University of Murcia, Murcia, Spain; ^6^Department of Anatomy, Histology and Neuroscience, School of Medicine, Autonomous University of Madrid, Madrid, Spain; ^7^Department of Anatomy and Cell Biology, College of Medicine, University of Illinois Chicago, Chicago, IL, United States

**Keywords:** diencephalon, prosomeres, mesomeres, SN, VTA, rhombomere, dopamine, nigrostriatal

## Abstract

During early developmental stages, the brain is divided into three primary regions: the forebrain (prosencephalon), the hindbrain (rhombencephalon), and the spinal cord. These regions are further segmented into transverse units called neuromeres, each with distinct molecular identities that guide their specialization through development. Such modular organization is evolutionarily conserved and shapes the structural and functional complexity of the brain. The substantia nigra (SN) and ventral tegmental area (VTA) are key midbrain regions involved in reward, motivation, and motor control. They contain dopamine-producing tyrosine hydroxylase (TH)-positive neurons, which are historically classified into three anatomical groups—A8 (retrorubral field), A9 (SN pars compacta), and A10 (VTA)—each with distinct anatomical and functional properties. Recent studies revealed further sub-regional organization along medial-lateral and anterior–posterior gradients, suggesting specialized roles tied to their developmental origins. This study uses the prosomeric framework to map the segmental distribution of TH-positive neurons within the SN and VTA across different mammalian species and developmental stages. Using a comparative analysis of rodent, non-human primate and human specimens, we were able to demonstrate that TH-positive neurons within the SN and VTA exhibit a multi-neuromeric organization, with neuronal populations distributed across the diencephalic prosomeres (dp1-dp3), the midbrain prosomeres (mp1-mp2) and the isthmic rhombomere (r0). It is therefore conceivable that such multi-neuromeric origin of TH-positive neurons within the SN and VTA likely influence the patterns of connectivity and functional specialization of the dopamine system.

## 1 Introduction

**Figure 1:**
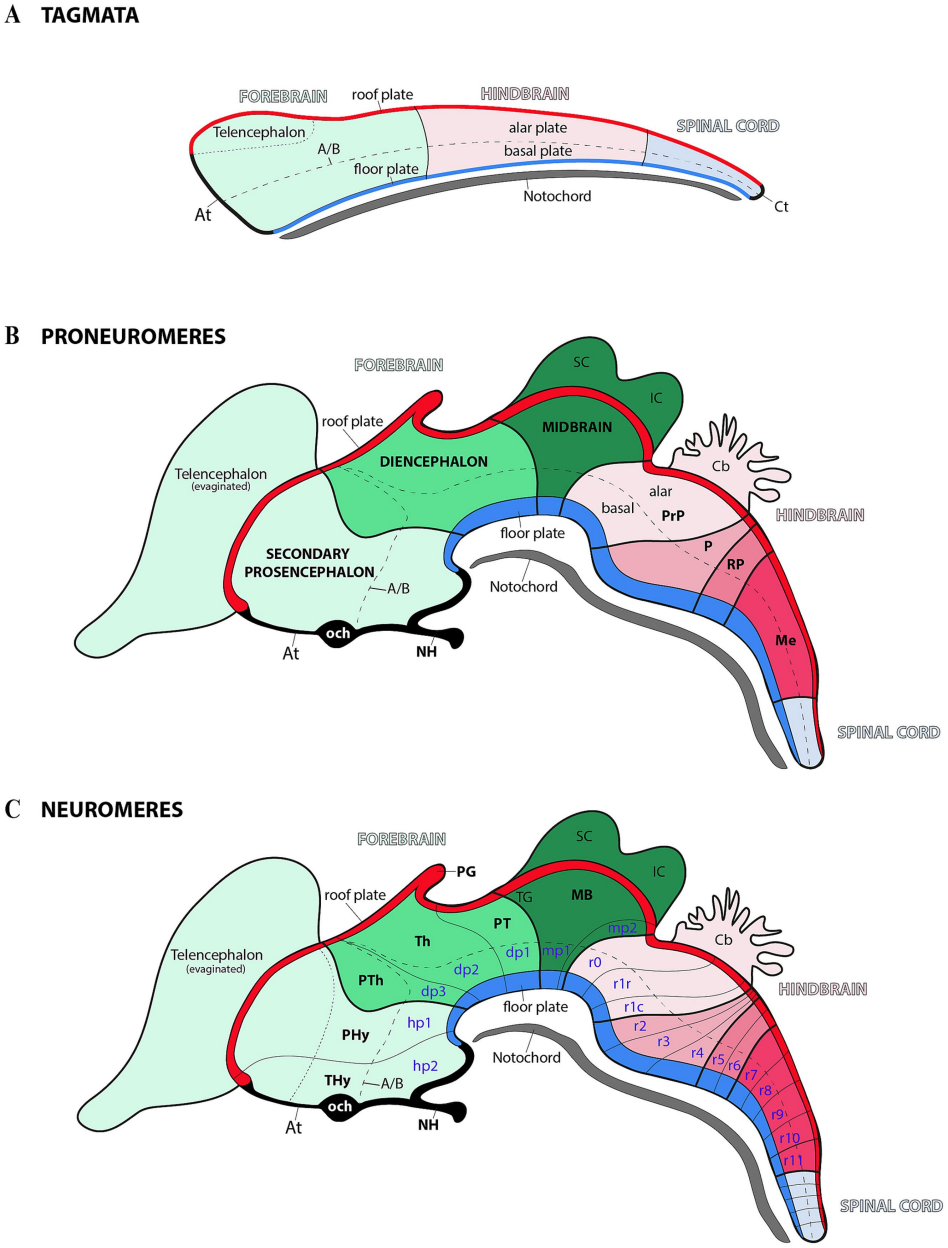
Schematic representation of progressive anterior–posterior (AP) neural regionalization during ontogeny, as delineated by the prosomeric model. The schemas primarily illustrate midline derivatives, with later developmental stages additionally incorporating some evaginated alar plate components (see below). **(A)** Following neural tube closure, three molecularly distinct anteroposterior compartments—termed ‘tagmata’—emerge: the forebrain (rostral), hindbrain, and spinal (caudal). Concurrently, the neural tube undergoes dorsoventral patterning, establishing four fundamental domains: roof, alar, basal, and floor plate. The acroterminal (At) and caudal terminal (Ct) domains demarcate the junction points where the alar and basal plates converge at the rostral and caudal ends of the neural tube. The notochord, represented extending along the full rostrocaudal axis, provides key signals for floor plate specification. Pale green, pale pink and pale blue serve as color codes for identifying forebrain, hindbrain and spinal cord territories, respectively. **(B)** During the proneuromeric stage, the forebrain differentiates into three primary subdivisions: (1) the secondary prosencephalon (rostral), (2) the diencephalon proper, and (3) the midbrain (caudal). Meanwhile, the hindbrain is regionalized into four distinct rostrocaudal partitions: the prepontine region (PrP, most rostral), followed by the pontine (P), retropontine (RP), and medullary territories (Me, most caudal). Both roof and floor plates become morphologically distinct during this stage, with the pineal gland (PG) emerging as a roof plate derivative. The telencephalon originates via evagination from the secondary prosencephalon’s dorsal alar plate, while the superior colliculus (SC) and inferior colliculus (IC) derive from the midbrain’s (MB) alar plate. Within the At domain, two structures are identifiable: the optic chiasm (och) and neurohypohisis (NH). The color code is a continuation of the initial scheme showing all forebrain derivatives in the green range, hindbrain derivatives in the pink range, and spinal cord-related derivatives in pale blue. **(C)** During the neuromeric stage, the developing brain reaches its definitive segmental units. The secondary prosencephalon forms two hypothalamo-prosencephalic prosomeres (hp1 and hp2, also called peduncular hypothalamus-PHy-and terminal hypothalamus-THy-, respectively), while the diencephalon proper develops three diencephalic prosomeres (dp1–dp3). The midbrain differentiates into two prosomeres (mp1 and mp2), and the hindbrain differentiates into 13 rhombomeres (r0–r11). A/B: alar-basal boundary; Cb: Cerebellum; OB: Olfactory bulb; PT: Pretectum; PTh: Prethalamus; TG: Tectal gray; Th: Thalamus.

During the early regionalization of the central nervous system (CNS), three primary regions (also known as tagmata) emerge: (1) prosencephalon or archencephalon (forebrain); (2) rhombencephalon (hindbrain); (3) spinal cord (Figure 1A) (Albuixech-Crespo et al., 2017; Ferran and Puelles, 2018; Puelles, 2018; Ferran et al., 2022). According to the prosomeric model, the CNS is organized along its anteroposterior axis into a series of transverse segments called neuromeres. Each neuromere acquires a distinct molecular identity through specific gene expression patterns, leading to the formation of specialized developmental units (for example, in the forebrain, the diencephalic prosomeres and midbrain prosomeres; but also in the hindbrain, rhombomeres) (Figures 1B,C) (Puelles and Rubenstein, 1993, 2003; Puelles and Rubenstein, 2015). This molecular patterning not only defines the structural and functional diversity of these regions but also underscores the evolutionary conservation of brain segmentation across vertebrate species. In this regard, the prosomeric model provides an unique framework that integrates molecular, developmental, and evolutionary processes critical for understanding how brain segmentation during development impacts the formation of the CNS across species (Puelles and Rubenstein, 1993, 2003; Puelles and Ferran, 2012; Puelles and Rubenstein, 2015; Albuixech-Crespo et al., 2017; Puelles, 2019).

The prosomeric model has been pivotal for understanding how distinct brain regions develop, differentiate, and establish functional connections (Puelles and Rubenstein, 1993, 2003; Puelles and Rubenstein, 2015). The notion is that the CNS is organized into modular units, each possessing distinct genetic, molecular, and functional identities. This modular architecture is prominent during embryonic development, where neuromeres act as foundational building blocks for the formation of the brain’s complex structures. These segmental units are not only anatomically defined but are also governed by specific genetic programs and signaling pathways that dictate their development and specialization (Puelles and Rubenstein, 1993, 2003; Puelles, 2009; Puelles and Ferran, 2012; Puelles and Rubenstein, 2015; Ferran, 2017; Puelles, 2019). The fact that the neuromeric organization is conserved across vertebrates highlights its role as a fundamental principle of brain development and its evolutionary expansion and diversification across species (Puelles and Rubenstein, 2003; Puelles, 2009; Puelles and Ferran, 2012; Puelles and Rubenstein, 2015; Ferran, 2017; Puelles, 2019).

The substantia nigra (SN) and the ventral tegmental area (VTA) are critical brain regions involved in motivation, reward processing, learning, and motor control (Hodge and Butcher, 1980; Morales and Margolis, 2017). Tyrosine hydroxylase (TH), the enzyme that converts L-tyrosine into L-DOPA, a precursor to dopamine (DA), is highly expressed in SN and VTA neurons. However, these cells largely lack noradrenergic and adrenergic markers such as dopamine-*β*-hydroxylase (DBH) and phenylethanolamine-N-methyltransferase (PNMT), indicating that TH-expressing cells in the SN and VTA are dopaminergic (Swanson and Hartman, 1975; Jaeger, 1986; Foster, 1998; Björklund and Dunnett, 2007; Morales and Margolis, 2017). In the 1960s, three major groups of dopamine (DA) neurons—A8 (located in the retrorubral field), A9 (located in the SN pars compacta), and A10 (located in the ventral tegmental area, VTA)—were identified and classified as belonging to the midbrain region (Dahlstroem and Fuxe, 1964; Björklund and Nobin, 1973; Swanson and Hartman, 1975; Fuxe et al., 1978; Jaeger, 1986; Foster, 1994; Foster, 1998; Björklund and Dunnett, 2007). Additionally, further studies have identified distinct subregions within the SNc and VTA, showing that certain properties are organized along medial-lateral and anterior–posterior gradients (Hanaway et al., 1970; Sanchez-Catalan et al., 2014; Morales and Margolis, 2017; Conrad et al., 2024).

Thus, the goal of the present study is to determine the developmental “diencephalo-meso-rhombencephalic” organization of TH-positive neurons within the SN and VTA across different mammalian species. Here we employed a comparative approach in combination with different histochemical markers to assess the multi-neuromeric distribution of TH-positive neurons across rodent, non-human primate and human brain samples. This study underscores the necessity of evaluating the potential functional consequences of the neuromeric organization of the SN and VTA, which is expected to provide insights on differential connectivity related with the specific neuromeric origin.

## 2 Materials and methods

The use of rats and mice in this study was approved by the Animal Research Ethics Committee (CEEA) of the University of Murcia (Authorization Numbers: A13200201, 869/2023) and conducted in compliance with Spanish regulations on animal research (RD 53/2013, Law 32/2007) and European Union directives (86/609/EEC). The study also adhered to the FORCED guidelines for housing and animal conditions (Garrigos et al., 2021). The human brain tissue was retrieved from Dr. Cavada’s anonymized brain archives at the Department of Anatomy, Histology, and Neuroscience of the School of Medicine of the Autónoma University of Madrid (Madrid, Spain; *n* = 1, neurotypical male, 58 year old); this tissue has been used in previous publications (García-Cabezas et al., 2007; Uceda-Heras et al., 2024) and its use for the present study was approved by the Ethics Committee for Research of Autonomous University of Madrid (Authorization CEI-104-2011) (García-Cabezas et al., 2007; García-Cabezas et al., 2023; Sancha-Velasco et al., 2023; Uceda-Heras et al., 2024). Two digital sagittal sections (0491 and 0535) of a *Macaca mulatta* brain, stained with Nissl, were obtained from BrainMaps: An Interactive Multiresolution Brain Atlas, MacBrainResource^1^ (Mikula et al., 2007).

### 2.1 Human samples

Human brain tissue from a neurotypical 58-year-old male, previously analyzed in multiple studies (García-Cabezas et al., 2007; Sancha-Velasco et al., 2023; Uceda-Heras et al., 2024), was obtained post-mortem and cut in brain slabs within the stereotaxic space of Talairach and Tournoux (García-Cabezas et al., 2023). Brain slabs (1 cm thick) were postfixed in 4% paraformaldehyde for 24–48 h and then cryoprotected in 30% phosphate-buffered sucrose. Then, small blocks containing the diencephalic and mesencephalic regions were separated from the slabs, cryoprotected in sucrose solutions, and coronally sectioned at 50 μm thickness using a freezing microtome. Consecutive sections were processed to examine the cytoarchitecture, myeloarchitecture, and chemoarchitecture of the brain tissue using cresyl violet staining, silver myelin staining (Gallyas, 1979), cytochrome oxidase histochemistry (Wong-Riley, 1979) and acetylcholinesterase histochemistry (Cavada et al., 1995), respectively. Tyrosine hydroxylase (TH) detection was performed using a mouse monoclonal anti-TH antibody (MAB318; 1:200–1:400; Chemicon, Temecula, CA) followed by a rabbit anti-mouse secondary antibody (AB240; 1:30; Chemicon) and incubation in mouse peroxidase-antiperoxidase (PAP) (PAP14; 1:600; Chemicon). The reaction was developed and intensified, with TH revealed using the sensitive glucose oxidase-diaminobenzidine (DAB)-nickel method (See details in Sánchez-González et al., 2005).

### 2.2 Rodents

Pregnant and adult Swiss mice, as well as pregnant, adolescent, and adult Sprague–Dawley (SD) rats, were obtained from the animal facilities at the University of Murcia. All animals were weighed and housed under identical conditions in standard cages (50 cm × 35 cm × 35 cm) with a 2–3 cm layer of dry cork bedding. Pregnant animals were sacrificed to obtain E12.5 mouse embryos and E13.5 rat embryos. The housing rooms were maintained at a temperature of 22–25°C with a relative humidity of 45–60%. Adolescent and adult animals had ad libitum access to a standard chow diet (ENVIGO, diet 2014, United States) and filtered water.

### 2.3 Rodent brain tissue processing

Swiss mouse and Sprague–Dawley rat brains were obtained and processed according to established protocols (Ferran et al., 2015a; Ferran et al., 2015b). The brains were perfused with a saline solution, followed by fixation with 4% paraformaldehyde in 0.1 M phosphate buffer (PB; pH 7.4). After extraction, the brains were fixed in 4% paraformaldehyde at 4°C for 16 h. Some brains were subsequently washed in phosphate-buffered saline (PBS) and cryoprotected in 15 and 30% sucrose solutions in 0.1 M PBS (pH 7.4). These brains were sectioned using a sliding microtome (Micron HM430, Thermo Scientific, United States) into 50 μm sagittal, horizontal, and transverse sections. Sections were collected as parallel or consecutive series on SuperFrost Plus slides (Menzel-Gläser, Braunschweig, Germany) and processed for hybridization and/or immunohistochemistry. Other brains were washed in PBS and embedded in 4% agarose (low electroendosmosis-EEO agarose; catalog No. 8008; Pronadisa, Spain) to obtain 100 μm vibratome sections. These sections were processed as free-floating samples for immunohistochemistry (Ferran et al., 2015a; Ferran et al., 2015b).

### 2.4 Rodents RT-PCR and cloning

A *Lim1* cDNA fragment was obtained through RT-PCR and cloned into a TA vector for subsequent RNA probe synthesis. Fresh postnatal mouse brain tissues were homogenized using the Precellys Evolution system (Bertin Technologies, France) with a single 20-s cycle at 6500 RPM in 2 mL tubes (CK14). Total RNA was extracted using the NZY Total RNA Isolation Kit (Nzytech, MB13402, Portugal) and treated with DNase I (Invitrogen, Cat. 18,068-015, United States). cDNA synthesis was performed using Superscript III reverse transcriptase (Invitrogen, Cat. 18080-044, Spain) and oligo dT-anchored primers. The resulting cDNA was used as a template for PCR amplification with Taq polymerase (Promega, Cat. M8305, Spain) and specific primers:

- Forward: 5´-GAGCGACAGGGCAATTAGAG-3´
- Reverse: 5´-GTCTGACACGCACACAACCT-3´

The amplified PCR products were cloned into the pGEM-T Easy Vector (Promega, Cat. A1360, Spain) and sequenced by ACTI (University of Murcia, Spain), yielding a 439 bp fragment (NCBI Accession Number: NM_008498.3, position 893–1,331).

### 2.5 Rodent tissue *in situ* hybridization

Brain sections for in situ hybridization, obtained using a vibratome, were collected on SuperFrost Plus slides and processed following previously published protocols (Ferran et al., 2015a; Ferran et al., 2015b). Linear cDNA templates for Gbx2, Lim1 and Pax6 were generated by PCR amplification of cloned fragments (see details for Gbx2 probe and Pax6 in Puelles et al., 2016) and (Puelles, 2019). Labeled sense and antisense RNA riboprobes were synthesized using digoxigenin-11-UTP (Roche, Lewes, United Kingdom) (Ferran et al., 2015a; Ferran et al., 2015b).

### 2.6 Rodent tissue immunohistochemistry

A detailed protocol for the immunohistochemical reaction has been described (Ferran et al., 2015a; Ferran et al., 2015b). Briefly, tissue sections were first treated with 0.3% hydrogen peroxide to inactivate endogenous peroxidases. Primary antibodies —mouse anti-NeuN (MAB377, Sigma-Aldrich, 1:4000), rabbit anti-Calbindin (CB38, Swant, 1:4000), and rabbit anti-TH (NB300-109, Novusbio, 1:200, Bio-Techne R&D Systems, Spain)—were incubated overnight at 4°C in rat and/or mouse sections. After washing, sections were incubated for 2 h with biotinylated secondary antibodies (goat anti-rabbit IgG (H + L) and goat anti-mouse IgG (H + L), Vector Laboratories, BA-1000-1.5 and BA-9200-1.5, 1:200). A streptavidin-peroxidase complex (Vectastatin-ABC kit, Vector Laboratories, United States; PK4000) was then applied for 1 h at room temperature. Finally, peroxidase activity was visualized using 0.03% 3,3′-diaminobenzidine (DAB, Sigma, St. Louis, MO, United States) with 0.003% hydrogen peroxide. Antibody specificity was confirmed by previous studies for TH (Bilbao et al., 2022), NeuN (Mullen et al., 1992) and CB (Caballero et al., 2014). Additional control experiments, omitting the primary antibody, showed no residual immunostaining.

### 2.7 Imaging

Processed in situ and immunohistochemistry sections were digitalized with a ScanScope CS digital slide scanner (Aperio Technologies, Vista, CA, United States). Size, contrast, brightness, and focus in the images were adjusted by applying Adobe Photoshop CS3. Figures were produced using Adobe Illustrator CS2 (Adobe Systems Inc., San Jose, CA, United States).

## 3 Results

### 3.1 Main anatomical landmarks identifying diencephalic, midbrain and rhombencephalic neuromeres

According to modern interpretations, the regionalization of the forebrain results in larger proneuromeric regions along the anteroposterior axis. These regions, from rostral to caudal, can be identified as the secondary prosencephalon, the diencephalon proper, and the midbrain (Figure 1B) (Albuixech-Crespo et al., 2017; Ferran and Puelles, 2018; Puelles, 2018; Ferran et al., 2022). The rostral proneuromere, known as the secondary prosencephalon, gives rise to two neuromeres: the peduncular hypothalamic prosomere (hp1) and the terminal hypothalamo-telencephalic prosomere (hp2) (Figure 1C) (Puelles et al., 2012a; Ferran et al., 2015c; Puelles and Rubenstein, 2015). Adjacent to these and within the diencephalon proper, there are three diencephalic prosomeres (Figure 1C, dp1–dp3). These neuromeres are also known as “pretectum” (dp1), “thalamus” (dp2), and “prethalamus” (dp3) (Puelles and Rubenstein, 1993; Rubenstein et al., 1994; Puelles et al., 2012b). Finally, in the most caudal proneuromere of the forebrain (often identified as the midbrain) there are 2 midbrain prosomeres (or mesomeres) (Figure 1C, mp1 and mp2) (Puelles E. et al., 2012c; Puelles, 2013; Watson and Puelles, 2017; Puelles, 2018, 2019; Puelles and Hidalgo-Sánchez, 2023). In the rhombencephalic region (hindbrain), the developing brain is organized into 13 distinct rhombomeres (Figure 1C, r0–r11) (Puelles, 2013; Puelles, 2018; Puelles and Hidalgo-Sánchez, 2023). This segmentation provides a developmental framework for understanding the organization of the forebrain and midbrain, including the spatial distribution of molecular markers from which structures like the SN and VTA emerge.

**Figure 2:**
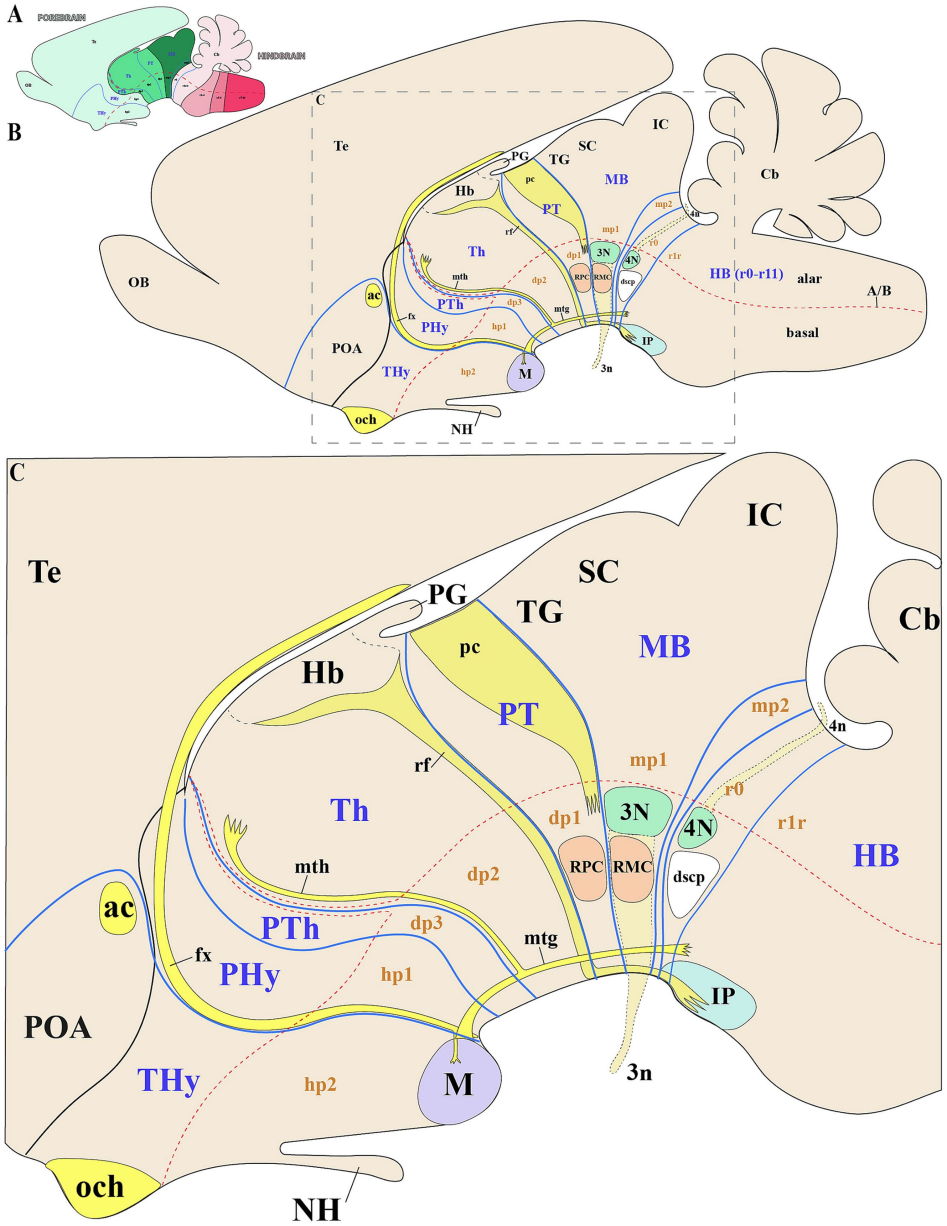
Schematic representation of key anatomical landmarks for identifying interneuromeric boundaries. **(A)** Schematic lateral view of a vertebrate mammal, with color-code identifying the derivatives from the forebrain (green range), hindbrain (pink range) and spinal cord (pale blue). **(B,C)** Schematic lateral view and enlargement of the brain of a vertebrate mammal (prosomeric model), showing subdivisions of the secondary forebrain (hp1, hp2), diencephalon (dp1–dp3), midbrain (mp1, mp2), and rhombomere r0. The alar plates of hp1 and hp2 are typically defined as the peduncular hypothalamus (PHy) or hypotalhamo-telencephalic prosomere 1 (hp1) and terminal hypothalamus (THy) or hypothalamo-telencephalic prosomere 2 (hp2), respectively. The dp1, dp2, and dp3 neuromeres are commonly distinguished by their major alar derivatives: the pretectum (PT), thalamus (Th), and prethalamus (PTh), respectively. The mp1 and mp2 neuromeres constitute the midbrain (MB), with the tectal gray (TG) superior colliculus (SC) and inferior colliculus (IC) arising as derivatives of mp1. Key tracts such as the optic chiasm (och), anterior commissure (ac), and fornix (fx) serve as important anatomical landmarks in the secondary prosencephalon. The fornix, which extends from the hippocampus to the mammillary body (M) and traverses the rostral part of hp1, demarcates the boundary between hp1 and hp2. From the mammillary body, two tracts arise: the mammillotegmental tract (mtg) and the mammillothalamic tract (mth). The mth projects dorsally through the basal and alar plates of dp2, passing near the rostral border of dp2 and dp3 before terminating in the anterior thalamic complex. Additionally, the habenulo-interpeduncular tract (retroflex tract) extends from the habenula to the interpeduncular nucleus in the caudal part of dp2, marking the boundary between dp1 and dp2. The dp1 prosomere contains the posterior commissure (pc), whose caudal border demarcates the dp1-midbrain boundary at the alar plate level. The parvocellular red nucleus (RPC), located in the dp1 basal plate, serves as a landmark for the rostral border of dp1, while its caudal adjacency to the magnocellular red nucleus (RMC) defines the dp1/midbrain boundary. In the midbrain basal plate, the oculomotor complex (3 N) and its nerve fibers (3n) mark the rostral midbrain limit. The trochlear nucleus (4N) and its nerve fibers (4n), the decussation of the superior cerebellar peduncle (dscp) and the interpeduncular nucleus (IP) delineate the isthmic rhombomere (r0), helping to identify the midbrain/isthmic boundary at the basal plate. Cb: Cerebellum; NH: Neurohypophysis; SC: Superior colliculus; POA: Preoptic area; Te: Telencephalon. Refer to the list for full anatomical abbreviations.

To assess whether the SN and VTA follow a polyneuromeric distribution, we will first define the key molecular and anatomical features of the midbrain and neighboring neuromeres (Figures 2A–C). The midbrain alar plate comprises four rostrocaudal rather than just the two classic colliculi: the tectal gray, superior colliculus, inferior colliculus (all within mp1), and alar preisthmus within mp2, but the basal plate contains the oculomotor nucleus complex in mp1 (Figures 1B,C, 2A–C) (Puelles E. et al., 2012c; Puelles, 2013; Watson et al., 2017; Puelles, 2018, 2019; Puelles and Hidalgo-Sánchez, 2023). The rostral boundary of the midbrain, which borders the diencephalic pretectal region (dp1), is discernible from early developmental stages in all vertebrates by the caudal limit of Pax6 expression in the alar plate (Ferran et al., 2007; Ferran et al., 2008; Ferran et al., 2009; Merchan et al., 2011; Morona et al., 2011, 2017; Brożko et al., 2022; Ferran and Puelles, 2024). Throughout later stages, anatomical landmarks can be used to identify this boundary. The pretecto-midbrain or diencephalon-midbrain boundary is defined by a plane extending from the dorsal roof plate to the ventral floor plate. This plane passes behind the posterior commissure in the alar plate, but anterior to the oculomotor complex (3 cranial nucleus) and between the red parvocellular and magnocellular nuclei, in the basal plate (the parvocelullar nucleus is in the basal plate of dp1 and the magnocellular nucleus in the basal plate of mp1) (Figure 2C) (Ferran et al., 2007; Ferran et al., 2009; Puelles E. et al., 2012c; Puelles, 2013; Watson et al., 2017; Puelles, 2018, 2019; Puelles and Hidalgo-Sánchez, 2023). The caudal midbrain boundary adjoins the isthmic region which contains the pathetic nucleus (4 cranial nucleus) and its associated nerve fibers (Watson et al., 2017; Puelles, 2019). Additionally, the interpeduncular nucleus which spans the isthmic (r0) and r1 rhombomeres, and the decusation of the superior cerebellar peduncle observed in the isthmic rhombomere help to identify the midbrain-isthmic boundary (dscp) (Figure 2C) (Lorente-Canovas et al., 2012). Key diencephalic landmarks can also help to identify interneuromeric boundaries. The retroflex tract (or habenulo-interpeduncular tract) marks the dp1-dp2 border, separating thalamus (dp2) from pretectum (dp1) (Figure 2C). This tract extends dorsoventrally from the habenula to the basal plate, passing through the caudal part of dp2 just rostral to dp1, but then along the basal plate to the interpeduncular nucleus (Figure 2C) (Ferran and Puelles, 2024). On the rostral side of the dp2, the mammillothalamic tract (mth), spanning from the mamillary body to the anterior thalamic complex, has a portion that defines the dp2-dp3 boundary (Figure 2C). Finally, the fornix tract (fx), coursing through rostral hp1, marks the hp1-hp2 boundary (Bilbao et al., 2022).

### 3.2 Multi-neuromeric organization of TH-positive neurons within the SN and VTA in rodents

**Figure 3:**
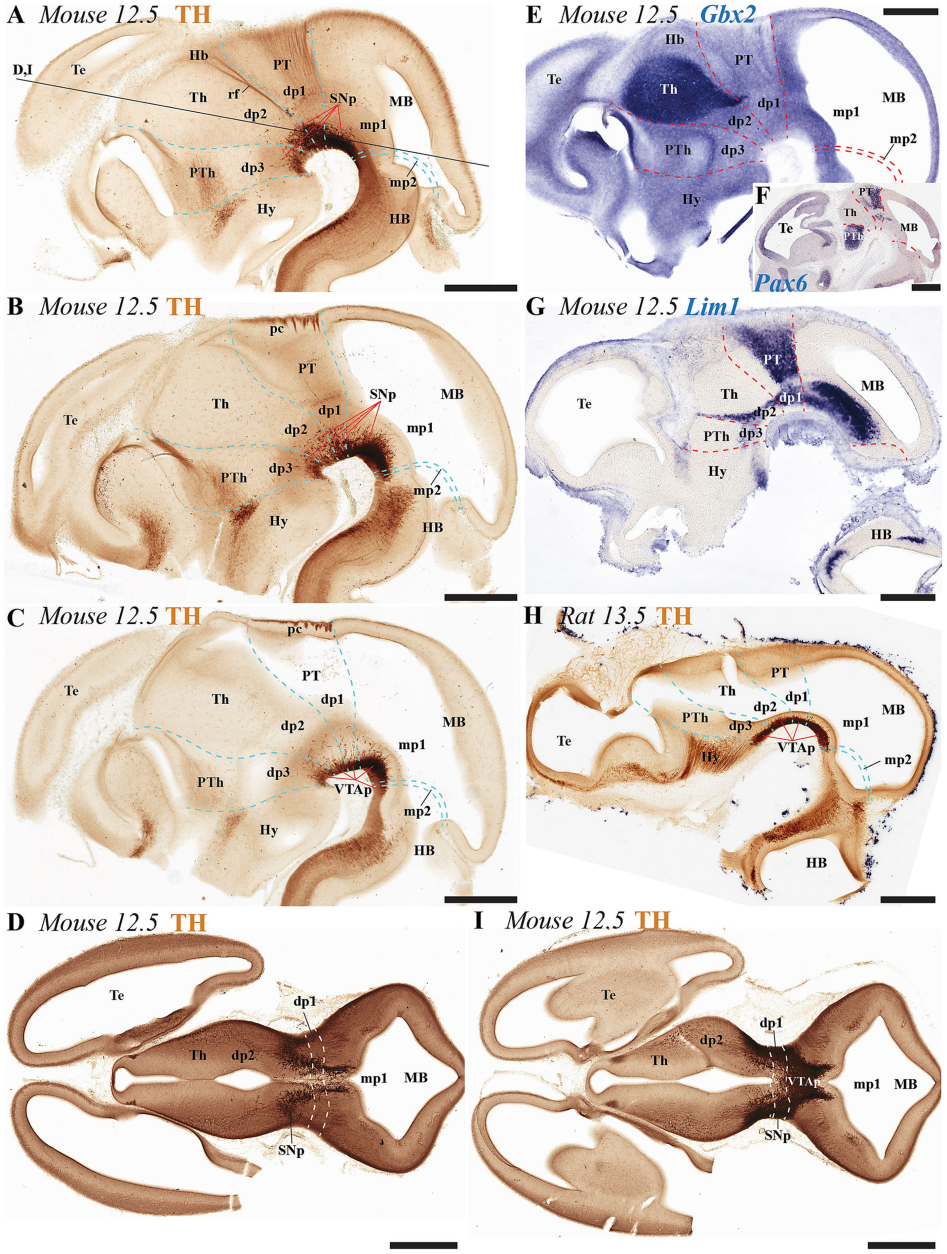
**(A–I)** Immunohistochemical and *in situ* hybridization analysis of early-stage embryonic mouse and rat brain vibratome sections. **(A–C)** Tyrosine hydroxylase (TH) immunostaining in three representative sagittal sections of mouse brain (lateral to medial progression). The substantia nigra (SN) and ventral tegmental area (VTA) primordia (SNp and VTAp) are undergoing differentiation and can be observed distributed across the diencephalic, midbrain and isthmic regions. Prosomeric boundaries (cyan dashed lines) are delineated using key anatomical landmarks, including the posterior commissure (pc) and retroflex tract (rf), as well as molecular markers (see gene expression patterns below). **(D,I)** Two representative horizontal sections (section plane indicated in panel **A**) demonstrate the localization of substantia nigra (SNp) and ventral tegmental area (VTAp) primordia within prosomeres dp1, dp2, and mp1. **(E–G)** Selected E12.5 mouse sagittal sections reveal distinct expression patterns of *Gbx2*, *Pax6*, and *Lim1* mRNA. *Gbx2* expression in the dp2 alar plate demarcates both its rostral border with dp3 and the caudal border with dp1. *Pax6* shows strong alar plate expression in p1, where its caudal boundary defines the dp1-midbrain border. *Lim1* exhibits restricted expression in the pretectal region, with: (1) its caudal boundary marking the dp1-midbrain border, and (2) its rostral expression domain in dp2 delineating the dp2-dp3 boundary **(H)** Representative sagittal section of rat brain at embryonic day E13.5 showing TH immunoreaction, with the ventral tegmental area primordium (VTAp) localized in the diencephalic-midbrain-isthmic region. Refer to the list for full anatomical abbreviations. Scale bar: 500 μm.

At early embryonic stages (E12.5 in mice and E13.5 in rats), the main neuromeric boundaries can be identified molecularly, aiding in the detection of TH-positive neurons that will later contribute to the adult substantia nigra pars compacta (SNc) and ventral tegmental area (VTA) (Figure 3). Cells belonging to the SNc primordium (SNcp) and VTA primordium (VTAp) are observed in both mice and rats within the diencephalic prosomeres (dp1-dp3), midbrain prosomeres (mp1-mp2) and the rostral hindbrain rhombomere (r0) (Figures 3A–I). Several studies have established molecular markers at this developmental stage to define interneuromeric boundaries in the diencephalon, midbrain and rhombencephalon (see previous section for details). For instance, *Gbx2* expression delineates the rostral p2/p3 boundary and the caudal p2/p1 boundary at E12.5 (Figure 3E). Also, *Pax6* is expressed in the alar plate of dp1 (pretectum), with its caudal border making the diencephalic-midbrain border (dp1-mp1) at E12-5 (Figure 3F). Finally, *Lim1* expression further aids in identifying both the diencephalic-midbrain boundary and the p2/p3 boundary across the alar and basal plates (Figure 3G).

**Figure 4:**
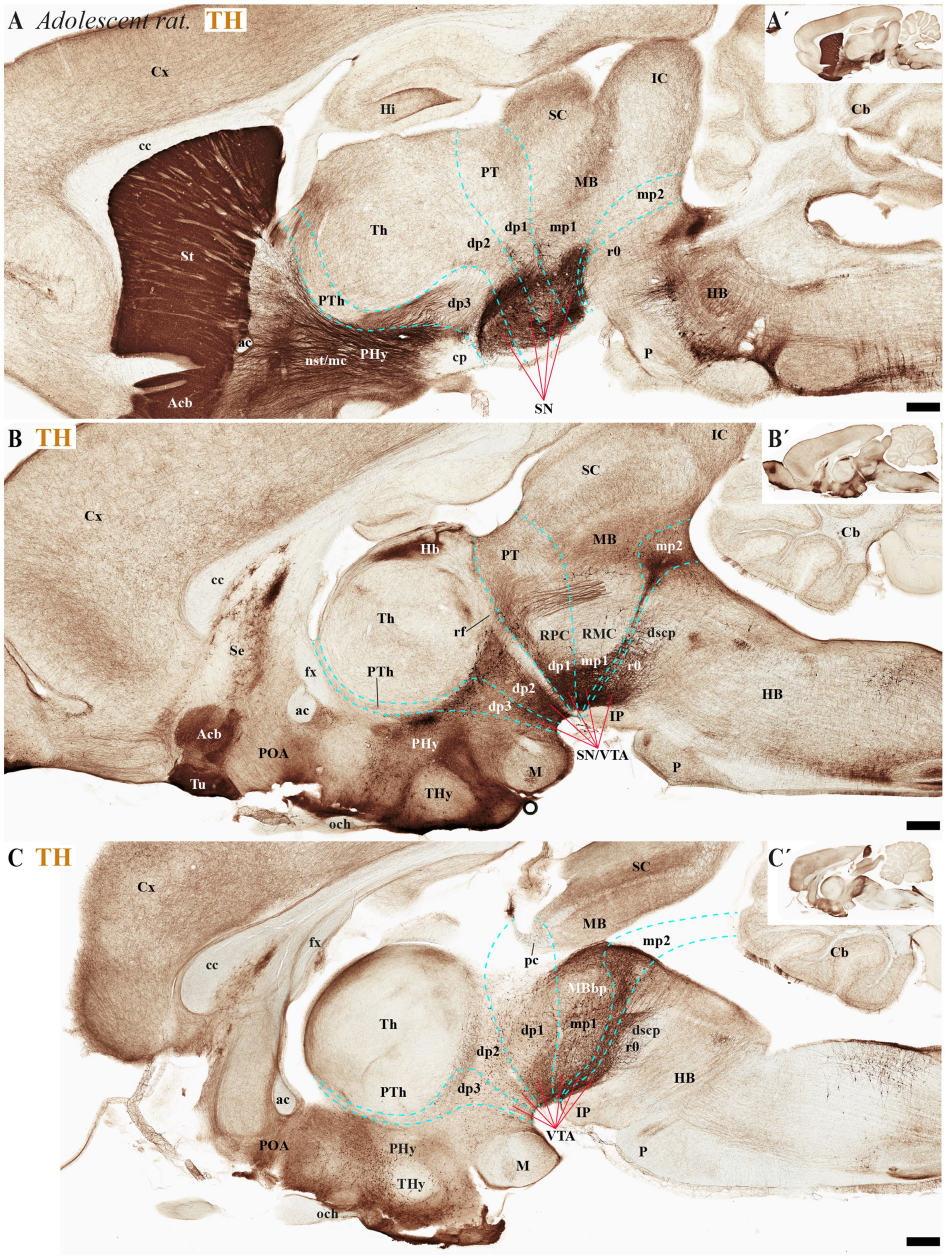
**(A–C´)** A lateral-to-medial series of high and low magnifications of sagittal sections from adolescent rats, processed for tyrosine hydroxylase (TH) immunohistochemistry, reveals the neuromeric distribution of substantia nigra (SN) and ventral tegmental area (VTA) dopaminergic populations. Diencephalic, midbrain, and rostral hindbrain neuromeric boundaries (cyan dashed lines) are delineated using key anatomical landmarks including the posterior commissure (pc) and retroflex tract (rf). The SN and VTA dopaminergic populations exhibit a multineuromeric distribution pattern during adolescence, spanning prosomeric domains from the diencephalon (dp1–dp3) through the midbrain (mp1-mp2) to the rostral hindbrain (r0 or isthmic rhombomere). The retroflex tract (rf) extends ventrally to the basal plate, creating a structural anteroposterior division in the SN and VTA. This separation occurs along a rostrocaudal axis, with a rostral portion located in dp2 prosomere and a caudal portion situated in dp1 prosomere **(B)**. Refer to the list for full anatomical abbreviations. Scale bar: 500 μm.

**Figure 5:**
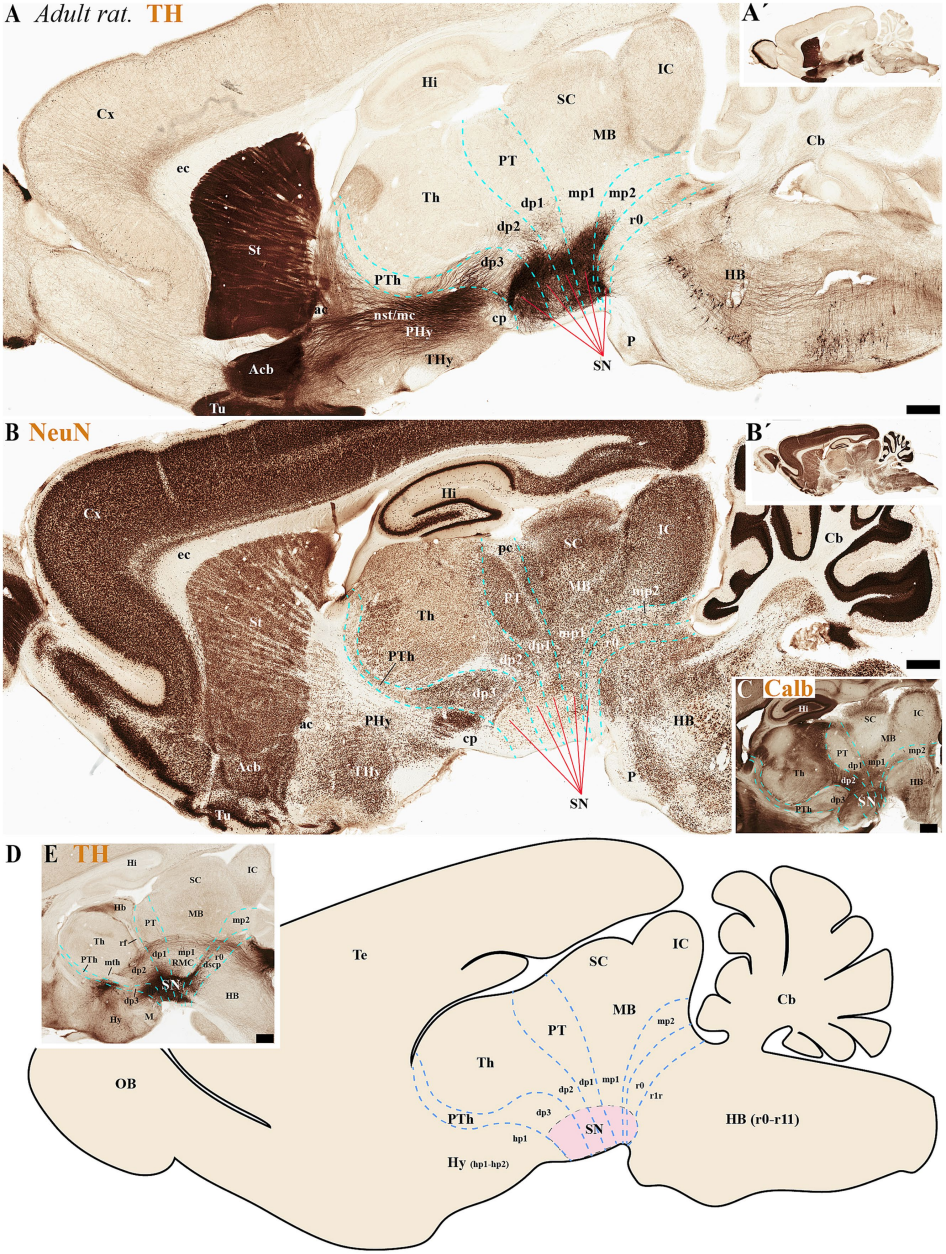
**(A–E)** A series of high and low magnifications of consecutive sagittal sections from adult rats, processed for tyrosine hydroxylase (TH), NeuN, and Calb immunohistochemistry, reveals the neuromeric distribution of substantia nigra (SN) dopaminergic populations. Calbindin (Calb) immunoreactivity in the thalamus serves as a reliable molecular marker for delineating two critical neuromeric boundaries: (1) its abrupt rostral diminution marks the transition to prethalamic territories, while (2) its sharp caudal termination defines the border with pretectal domains **(C)**. Complementing these boundaries, distinct tracts provide structural landmarks for adjacent prosomeric divisions: the retroflex tract (rf) demarcates the dp1-dp2 interface, while the posterior commissure (pc) forms a transverse fiber bundle that establishes the dp1-midbrain boundary **(E)**. The schematic diagram presents a lateral perspective of the adult rat brain, illustrating the multineuromeric organization of the substantia nigra (SN) across three domains: (1) the diencephalon (prosomeres dp1-dp3), (2) midbrain (mesomeres mp1-mp2), and (3) isthmic region (rhombomere r0). This spatial distribution reveals how dopaminergic neuron populations maintain their embryonic compartmentalization into adulthood **(D)**. Refer to the list for full anatomical abbreviations. Scale bar: 500 μm.

**Figure 6:**
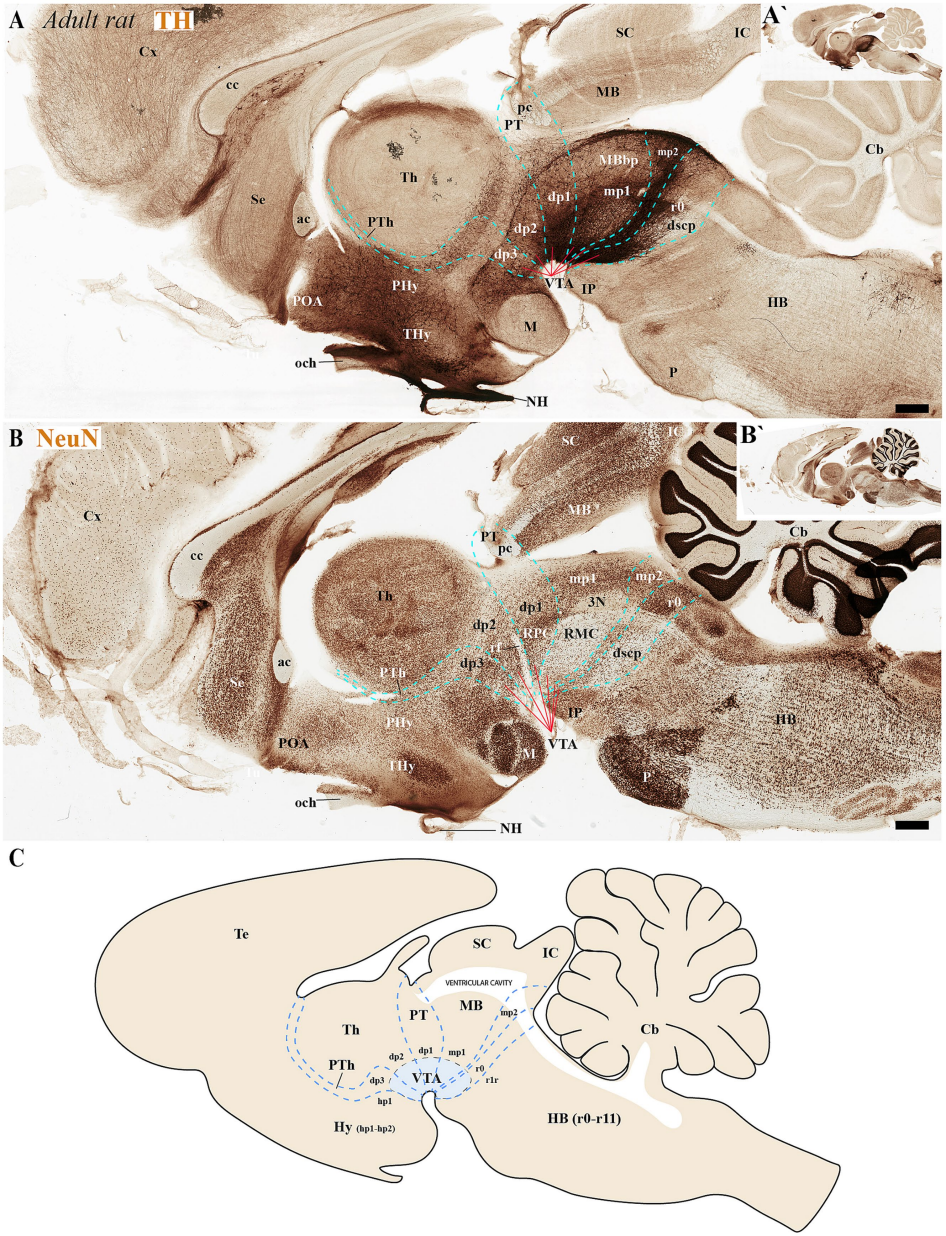
**(A–C)** Through analysis of consecutive sagittal sections from adult rats, positioned medial to those shown in Figure 5 and processed for tyrosine hydroxylase (TH) and NeuN immunohistochemistry, we reveal the neuromeric distribution of ventral tegmental area (VTA) dopaminergic populations. Examination at both high and low magnification demonstrates the precise organization of TH-positive neurons within specific neuromeric domains, while NeuN labeling in consecutive sections confirms their mature neuronal identity. These medial sections particularly highlight the spatial relationship of VTA dopaminergic cells with different neuromeric compartments. The schematic diagram presents a lateral perspective of the adult rat brain, illustrating the multineuromeric organization of the ventral tegmental area (VTA) **(C)**. Refer to the list for full anatomical abbreviations. Scale bar: 500 μm.

**Figure 7:**
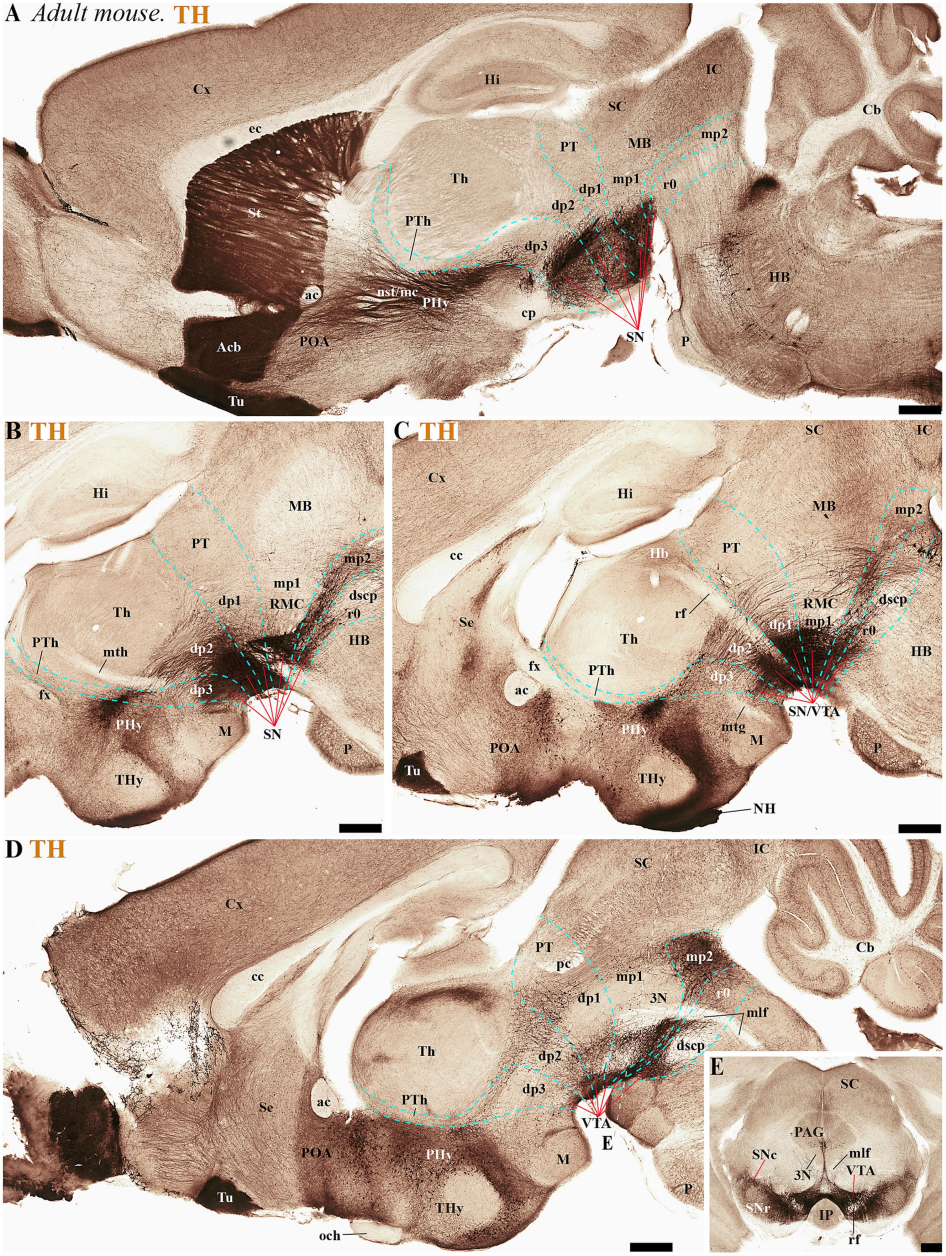
**(A–D)** A lateral-to-medial series of high and low magnifications of sagittal sections from adult mice, processed for tyrosine hydroxylase (TH) immunohistochemistry, reveals the neuromeric distribution of substantia nigra (SN) and ventral tegmental area (VTA) dopaminergic populations. Three key tracts serve as anatomical landmarks for defining interprosomeric boundaries: the mamillothalamic tract (mth) **(B)** marks the p2/p3 boundary through its pathway connecting mammillary bodies to the anterior thalamus, retroflex tract (rf) **(C)** identifies the p1/p2 border via its trajectory from habenula to interpeduncular nucleus and posterior commissure (pc) **(D)** delineates the dp1-midbrain transition. **(E)** Transversal section through the midbrain region (see plane of section in **D**) showing the distribution of three functionally distinct brain structures: the substantia nigra compacta (SNc), substantia nigra reticular (SNr) and ventral tegmental area (VTA). Refer to the list for full anatomical abbreviations. Scale bar: 500 μm.

During the adolescent and adult stages of rodents (mice and rats) TH-positive neurons within the SN and VTA persist in locations similar to those observed in early developmental stages. These neurons remain situated in the basal plate across all diencephalic prosomeres (dp1-dp3), midbrain prosomeres (mp1-mp2), and the rostral rhombomere (r0) (Figures 4A–C, 5A–E, 6A–C, 7A–E). Some anatomical landmarks, as described in the previous section, help delineate the interneuromeric boundaries. The mammilo-thalamic tract, situated in the rostral part of the diencephalic prosomere 2 (dp2), serves as a landmark for the dp2/dp3 boundary. This boundary demarcates distinct TH-positive neuronal populations belonging to the SNc and VTA and located in the basal plate of dp2 and dp3 neuromeres (Figures 5E, 7B). Just caudal to the retroflex tract (rf) lies the boundary between the dp1 and dp2 neuromeres. This tract extends from the dorsal alar plate to the ventral basal plate, dividing the SNc and VTA TH-positive neurons into a rostral dp2 group and a caudal dp1 group (Figures 4B, 5E, 6B, 7C). The border between diencephalon and midbrain (di-mb or dp1-mp1) lies caudal to the posterior commissure (alar plate; Figures 4C, 5B, 6A,B, 7D), and rostral to the oculomotor complex (3N) (basal plate; Figure 6B). In the basal plate, the border separating the parvocellular (RPM, dp1) and magnocellular (RMN, mp1) red nuclei (Figure 6B), delineates TH-positive neurons within the SN and VTA into dp1 and mp1 neuromeres (Figures 5E, 6B). The RMC caudal border indicates the location of mp2 (Figure 6B). The location of the decusation of the superior cerebellar peduncle (dscp) and the interpeduncular nucleus in r0 recognizes in their anterior parts the boundary between midbrain (mp2) and isthmic region (r0) (Figures 4B,C, 6A,B, 7B–D).

### 3.3 Multi-neuromeric organization of TH-positive neurons within the SN and VTA in primates

**Figure 8:**
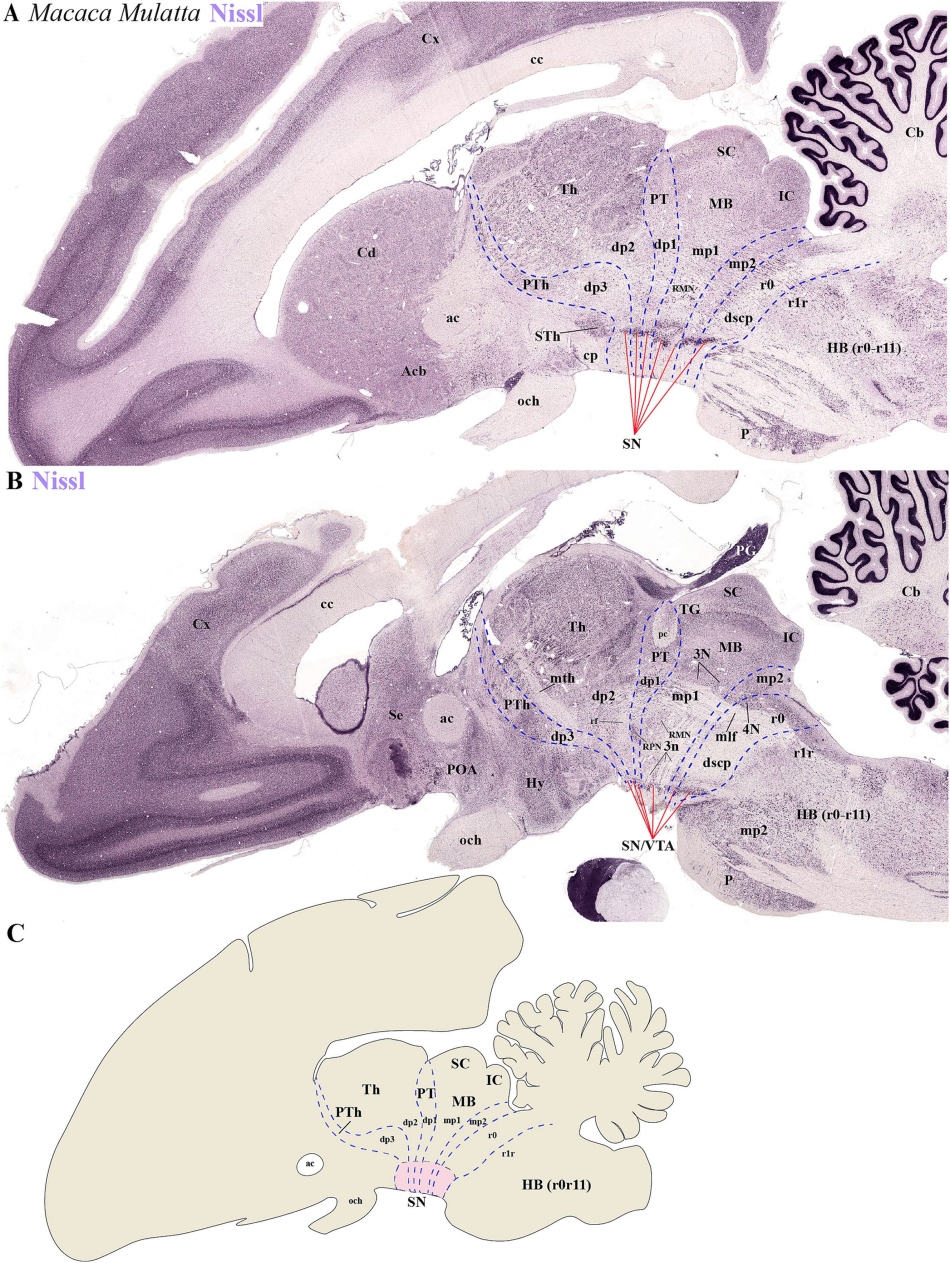
**(A–C)** Representative sagittal sections from lateral to medial planes in *Macaca mulatta*, along with a schematic representation, demonstrate the neuromeric organization of substantia nigra (SN) and ventral tegmental area (VTA) populations through Nissl staining. **(A)** The subthalamic nucleus (STh) serves as a critical anatomical landmark for identifying neuromeric boundaries in lateral sections. Its distinct position clearly demarcates the rostral termination of the substantia nigra (SN) while simultaneously defining the border between diencephalic prosomere dp3 and hypothalamic prosomere hp1. This topographical relationship remains consistent across mammalian species, making the STh an essential reference point for both developmental studies and adult neuroanatomical mapping. **(B)** In medial sagittal sections, three major axonal pathways serve as essential anatomical landmarks for identifying interprosomeric boundaries: the mammillothalamic tract (mth), which defines the p2/p3 boundary through its connection between mammillary bodies and anterior thalamic nuclei; the retroflex tract (rf), marking the p1/p2 border and the posterior commissure (pc), delineating the p1-mp1 transition at the diencephalic-midbrain interface. Complementary to these white matter tracts, the paired parvocellular and magnocellular red nuclei in the basal plate provide additional guidance for recognizing the p1-midbrain boundary, as does the rostral margin of the oculomotor nucleus (3N) and its emerging nerve fibers (3n). Finally, location of 4 N and decusation of superior cerebellar peduncle (dscp) help to identify the ro/midbrain boundary **(C)** Schematic diagram showing a lateral perspective of the adult primate *Macaca Mulatta* brain, illustrating the multineuromeric organization of the substantia nigra (SN). Refer to the list for full anatomical abbreviations.

To determine the distribution of SN and VTA neuronal groups in the primate *Macaca mulatta*, we analyzed two Nissl-stained sagittal sections obtained from *BrainMaps.org* . The selected sections, which pass through SN or SN/VTA regions, reveal their distribution in the basal plate of distinct neuromeres, including diencephalic (dp1-dp3), midbrain (mp1-mp2), and rostral hindbrain (r0) segments (Figures 8A–C). The most rostral group is located in the basal plate of dp3, caudal to the subthalamic nucleus (Sth) (Figure 8A). Key anatomical landmarks help identifying interprosomeric boundaries, such as the mammillothalamic tract (mth) between dp2 and dp3, and the retroflex tract (rf), marking the boundary between dp1 and dp2 (Figure 8B). At the alar plate level of dp1 (pretectal region), the posterior commissure (pc) is visible, with its caudal border demarcating the dp1/mp1 (diencephalon-midbrain) boundary (Figure 8B). Additionally, the parvocellular and magnocellular red nuclei serve as markers for the dp1/mp1 border (Figure 8B). The sections also highlight the oculomotor complex (3 N) in the midbrain basal plate and its fibers (3n) extending toward the interpeduncular fossa (Figure 8B). Both 3 N and 3n indicate the dp1/mp1 border rostrally and the mp1/mp2 border caudally. The mp2/r0 border can be recognized by the rostral parts of the decusation of the superior cerebellar peduncle (dscp) and the IP nucleus (Figures 7A,B). This preliminary analysis suggests that a significant population of SN and VTA neurons is situated in the most rostral rhombomere (r0) (Figures 8A–C).

**Figure 9:**
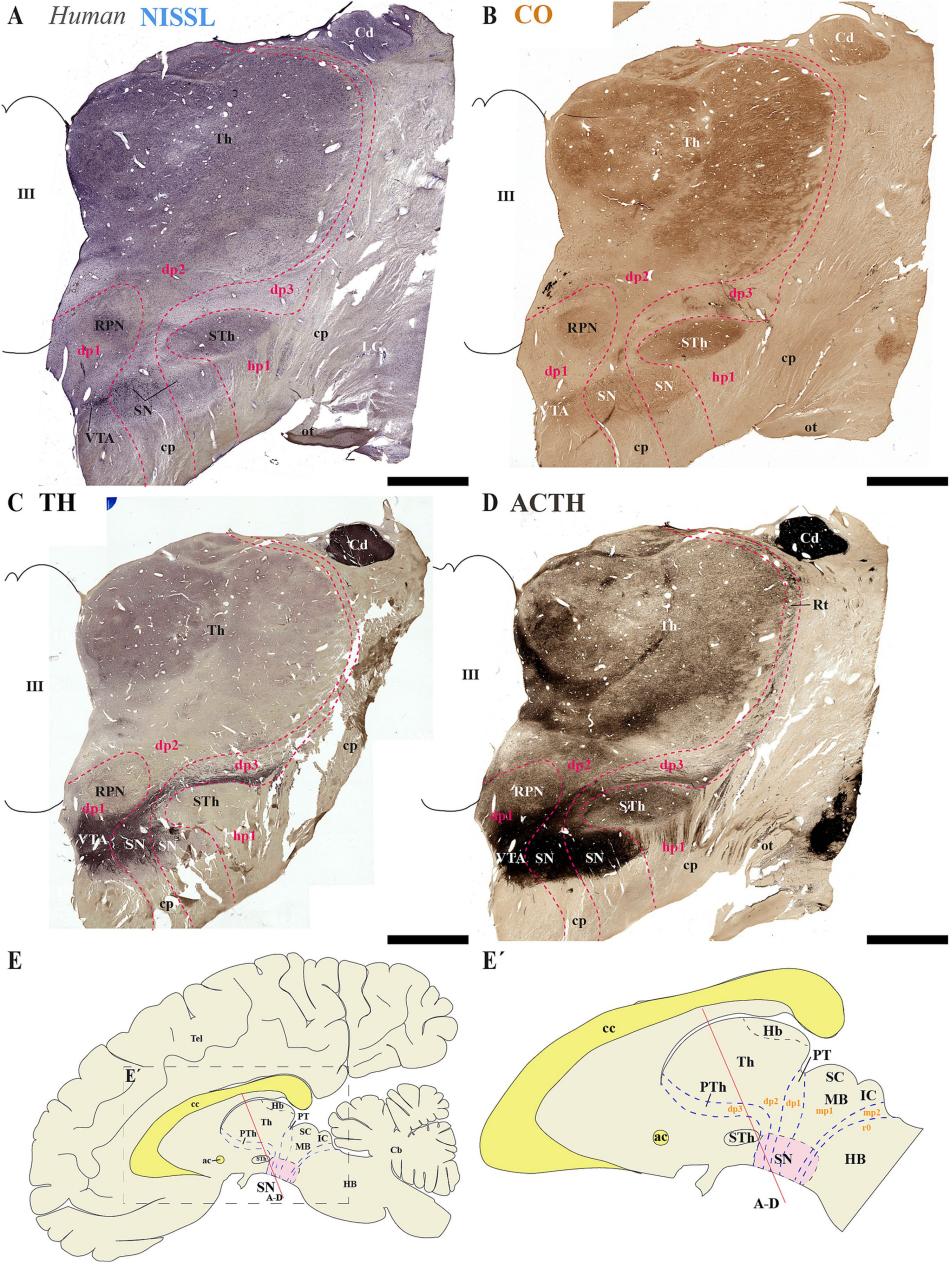
**(A–D)** A series of representative human brain coronal sections, oriented according to the lateral schematic view **(E,E´)**, were selected that traverse the diencephalic prosomeres dp1-dp3 and hypothalamic prosomere hp1. Consecutive sections were processed for Nissl staining–revealing cytoarchitectonic boundaries-, Cytochrome oxidase (CO) histochemistry–marking metabolic activity patterns-, Tyrosine hydroxylase (TH) immunohistochemistry–identifying dopaminergic populations-and Acetylcholinesterase (AChE) staining–delineating cholinergic pathways. The subthalamic nucleus (STh, hp1 derivative) and reticular nucleus (Rt, dp3 derivative) serve as anatomical landmarks that delineate the boundary between hypothalamic prosomere hp1 and diencephalic prosomere dp3 **(C)**. This spatial relationship reveals a distinct portion of the substantia nigra (SN) that extends from dp3 to directly about the hp1 border **(C)**. The parvocellular red nucleus (RPN) serves as a definitive marker for the p1 basal plate, delineating the ventral tegmental area (VTA) territory within this prosomere **(C)**. Furthermore, the expanded thalamic nuclei reveal the alar plate organization of dp2, indicating a distinct population of substantia nigra (SN) positioned at the interface between dp1 and dp3 domains **(C)**. The schematic lateral view of the human brain illustrates the multineuromeric organization of the substantia nigra (SN), with distinct populations distributed across diencephalic (dp1–dp3), midbrain (mp1) and isthmic (r0) prosomeres **(E,E’)**. Refer to the list for full anatomical abbreviations. Scale bar: 500 μm.

**Figure 10:**
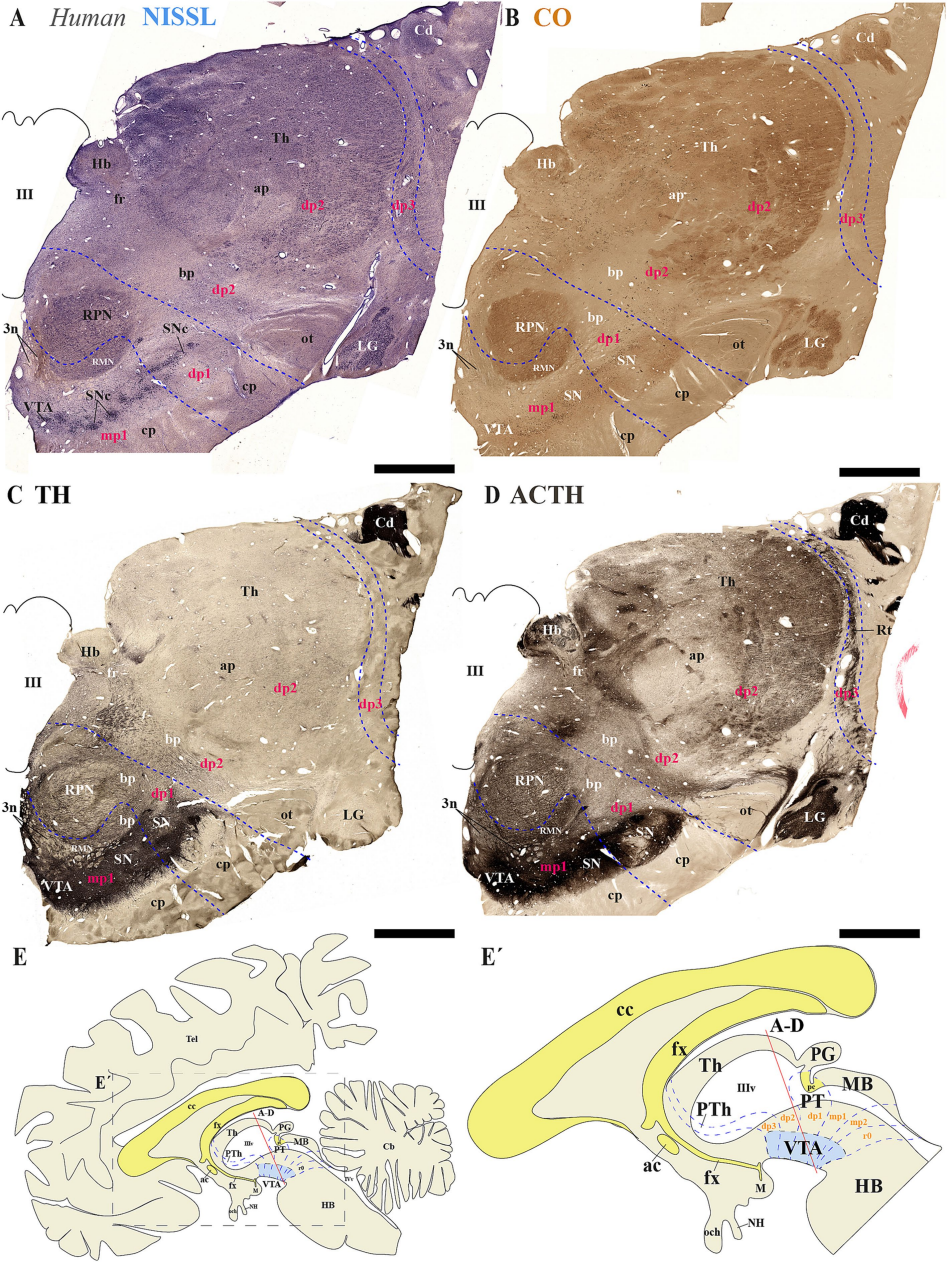
**(A–E´)** A series of representative human brain coronal sections, oriented according to the lateral schematic view **(E)**, were selected that traverse the diencephalic prosomeres dp1-dp3 and midbrain prosomere 1 (mp1). Consecutive sections were processed for Nissl staining–revealing cytoarchitectonic boundaries-, Cytochrome oxidase (CO) histochemistry–marking metabolic activity patterns-, Tyrosine hydroxylase (TH) immunohistochemistry–identifying dopaminergic populations-and Acetylcholinesterase (AChE) staining–delineating cholinergic pathways. The reticular nucleus (Rt) precisely demarcates the alar plate of diencephalic prosomere dp3 and its boundary with the thalamic alar plate (dp2). Within the dp2 alar plate, two prominent structures emerge: (1) the habenular complex (Hb) medially, and (2) the lateral geniculate nucleus (LG) positioned ventrolaterally adjacent to the optic tract (ot). In the basal plate, the parvocellular and magnocellular red nuclei serve as key landmarks for identifying the dp1 territory, containing substantia nigra (SN) populations, and the mp1 domain, harboring both SN and ventral tegmental area (VTA) neuronal groups. This organization reveals a conserved topological relationship between neuromeric boundaries and multineuromeric dopaminergic groups. The schematic lateral view of the human brain illustrates the multineuromeric organization of the substantia nigra (SN), with distinct populations distributed across diencephalic (dp1–dp3), midbrain (mp1) and isthmic (r0) prosomeres **(E,E’)**. Refer to the list for full anatomical abbreviations. Scale bar: 500 μm.

Next, we analyzed human brain sections stained with Nissl, cytochrome oxidase (CO), tyrosine hydroxylase (TH) and acetylcholinesterase (ACTH) histochemistry, focusing on regions encompassing through the subthalamic nucleus. These sections allowed us to examine the spatial relationships between de SN and VTA with diencephalic prosomeres (Figures 9A–E´). Additionally, we studied more caudal sections to assess their association with the midbrain prosomere 1 (mp1) (Figures 10A–E´). These coronal sections were cut perpendicular to a reference plane defined by two anatomical landmarks: the superior border of the anterior commissure (ac) and the inferior border of the posterior commissure (pc) (Figures 9E, E´, 10E,E´). Rostral sections traversing the Sth, reveal its location within the hypothalamo-telencephalic prosomere 1 (hp1), which is further characterized by cerebral peduncle (cp) fibers coursing along its surface (Figures 9A–D). Adjacent to hp1, dp3 is identified by the presence of the reticular nucleus in its alar plate (Figure 9D). The dp3 alar plate continues to its basal plate bordering the subthalamic nucleus and extending to the surface at the cp (Figures 9A–D). The extensive thalamic nuclear region (Th, dp2) and parvocellular red nucleus (RPN, dp1) are readily identifiable through distinct anatomical landmarks, confirming the presence of both neuromeres in these sections. Based on this anatomical analysis, we concluded that the SN primarily localizes to dp3 and dp2 prosomeres, while VTA and possibly a small population of SN neurons occupy dp1 (Figures 9A–D). In the analyzed caudal sections, the dp3 prosomere is clearly delineated by the presence of the reticular nucleus within its alar plate (Figure 10D). The alar plate of dp2 prosomere is identified by the presence of the extensive thalamic (Th) complex, the habenular region (Hb), and the lateral geniculate nucleus (LG) (Figures 10A–D). At the basal plate level, we observe an important distinction: while the parvocellular red nucleus marks dp1 territory, the magnocellular red nucleus identifies mp1 (midbrain prosomere 1). This organization reveals that: (1) a distinct SN population resides within dp1 and (2) both SNc and VTA neuronal groups are predominantly located in mp1 at this level of section. The midbrain mp1 prosomere is further defined by the path of oculomotor nerve fibers (3n) traversing this region (Figures 10A–D). These findings demonstrate that in adult humans, both the VTA and SN span multiple neuromeric domains, including diencephalic prosomeres (dp1-dp3), midbrain prosomeres (mp1-mp2) and the rostral hindbrain rhombomere (r0) (Figures 9E, 10E´).

## 4 Discussion

Our study employed the prosomeric framework to reveal that TH-positive neurons within the SN and VTA originate from multiple neuromeres beyond those from the midbrain (Figures 5D, 6C, 8C, 9E, 10E). By using conserved neuroanatomical landmarks that reliably delineate inter-neuromeric boundaries across species (Puelles and Rubenstein, 1993, 2003; Puelles and Rubenstein, 2015), we found that the multi-neuromeric organization of TH-positive neurons within the SN and VTA includes neuronal populations distributed across the diencephalic prosomeres (dp1-dp3), the midbrain prosomeres (mp1-mp2) and the isthmic rhombomere (r0).

Classical neuroanatomical studies have traditionally classified the substantia nigra (SN) and ventral tegmental area (VTA) as core components of the midbrain (Olszewski and Baxter, 1954; McRitchie et al., 1995; Paxinos and Huang, 1995). This classification emerged from early cytoarchitectonic studies that positioned these structures within the midbrain tegmentum, and was further supported by their well-documented connectivity with basal ganglia structures (Carpenter and Peter, 1972). The identification of the dopaminergic nature of these nuclei through histofluorescence techniques (Dahlstroem and Fuxe, 1964), became a defining characteristic that consolidated their midbrain location in standard neuroanatomical atlases (Paxinos and Watson, 1982; Swanson, 1992). Through the late 20th century, this conventional perspective remained dominant, with major neuroanatomical textbooks consistently describing the SN and VTA as mesencephalic structures (Brodal, 1981; Nieuwenhuys, 1985; Parent, 1996), despite emerging evidence of their complex developmental origins. However, advances in developmental neurobiology, particularly the segmental neuromeric model, have revealed a more complex and nuanced organization of these structures. According to this model, the SN and VTA are not confined to a single neuromere but instead exhibit a multi-neuromeric origin, extending across the diencephalo-meso-isthmic complex. This domain encompasses a continuum from the rostral diencephalon (dp1-dp3) through the midbrain (mp1-mp2) and into the isthmic hindbrain region (r0) (Medina et al., 1994; Puelles and Medina, 1994; Marın et al., 1998; Verney et al., 2001; Marín et al., 2005; Puelles E. et al., 2012c; Puelles et al., 2012a; Puelles et al., 2012b; Puelles, 2013; Puelles, 2019). Studies across a wide range of species, including amphibians, chickens, mice, and human embryos, have provided compelling evidence that TH-positive neurons are primarily located within several neuromeric territories during early development (Medina et al., 1994; Puelles and Medina, 1994; Marın et al., 1998; Verney et al., 2001; Marín et al., 2005; Puelles E. et al., 2012c; Puelles et al., 2012a; Puelles et al., 2012b; Puelles, 2013; Puelles, 2019). This revised framework, which is aligned with gene expression patterns, axonal projections and embryological origin, suggest that TH-positive neurons within the SN and VTA arise from multiple progenitor domains rather than a single mesencephalic segment (Puelles E. et al., 2012c; Puelles et al., 2012b; Puelles, 2013; Puelles, 2019), and challenges the traditional midbrain-centric view. Such paradigm shift has significant implications for understanding the ontogeny, connectivity and functional organization of SN and VTA TH-positive neurons in vertebrates, which form part of a broader developmental field that integrates diencephalic, mesencephalic and isthmic influences (Puelles, 2013; Puelles, 2018, 2019).

The multi-neuromeric organization of the SN and VTA is likely to influence the interpretation of their afferent and efferent connectivity patterns. Emerging evidence suggests each partition within these multi-neuromeric structures exhibit specialized connectivity profiles, which may underline their diverse functional roles in reward, aversion, and motor control. For instance, the lateral habenula preferentially targets the medial posterior VTA (caudal) driving dopamine neurons that project to the medial prefrontal cortex (Lammel et al., 2012). This pathway plays a crucial role in aversion-related behaviors and reward prediction error signaling (Matsumoto and Hikosaka, 2007; Proulx et al., 2014). Conversely, rostral VTA may receive inputs from the lateral hypothalamus and preferentially innervates the nucleus accumbens, reinforcing reward-seeking behaviors (Beier et al., 2015; Nieh et al., 2015; Nieh et al., 2016). The VTA contains heterogeneous populations of dopamine, GABA, and glutamatergic neurons, each with distinct efferent and afferent pathways (Morales and Margolis, 2017; Conrad et al., 2024). In the case of the SN, the rostral neuronal population sends collaterals to both striatum and prefrontal cortex, while the caudal population projects more selectively to the striatum, suggesting a developmental or functional subdivision (Prensa and Parent, 2001; Matsuda et al., 2009). By means of intersectional genetic approaches, it was also revealed that transcriptional subtypes of SN and VTA dopaminergic neurons correlate with their striatal projection targets (Poulin et al., 2018). Such organizational complexity underscores the need for refined anatomical and functional studies to fully elucidate the role of the SN and VTA neuromeric partitions in neural circuits.

Traditionally, the development of the nervous system is believed to follow a columnar organization parallel to the longitudinal axis. Specifically, it divides the brain into four functional columnar domains (i.e., somatic motor, visceral motor, visceral sensory, and somatic sensory). However, the proposed columnar organization fails to explain how each functional domain differentiates into distinct nuclei (Puelles and Rubenstein, 1993, 2003; Puelles and Rubenstein, 2015; Puelles, 2018, 2019; Bilbao et al., 2022). On the other hand, the prosomeric framework takes into account the dynamic temporo-spatial patterns of gene expression to reveal different functional segmentations with unique genetic identity along the antero-posterior axis (Puelles and Rubenstein, 2015; Puelles, 2018, 2019). In this regard, it is conceivable that the heterogeneity of TH-positive neurons has a developmental origin with each neuromeric population exhibiting distinct molecular and functional characteristics. Such interpretation can be extended to TH-positive neuronal populations in primates as evidence supporting a similar multi-neuromeric origin delineated by the same anatomical landmarks has been reported in the developing human brain (Puelles and Verney, 1998). In conclusion, the multi-neuromeric organization of TH-positive neurons within the SN and VTA highlights the importance of considering neuromeric-regional specificity when mapping their unique connectivity patterns that typically begins at early stages of brain development.

## Data Availability

The raw data supporting the conclusions of this article will be made available by the authors, without undue reservation.

## Footnotes

^1^ www.brainmaps.org

## Glossary

3N: Oculomotor complex
3n: Third nerve fibers
4N: Pathetic nucleus
4n: Fourth nerve fibers
A/B: Alar-Basal boundary
ac: Anterior commissure
Acb: Accumbens nucleus
ap: Alar plate
At: Acroterminal
bp: Basal plate
Cb: Cerebellum
cc: Corpus callosum
Cd: Caudate (Striatum)
cp: Cerebral peduncle
Cx: Cortex
Ct: Caudal terminal
dp1: Diencephalic prosomere 1
dp2: Diencephalic prosomere 2
dp3: Diencephalic prosomere 3
dscp: Decussation of the superior cerebellar peduncle
ec: External capsule
fx: Fornix
HB: Hindbrain
Hb: Habenula
Hi: Hippocampus
Hy: Hypothalamus
hp1: Hypothalamo-telencephalic prosomere 1
hp2: Hypothalamo-telencephalic prosomere 2
IC: Inferior colliculus
IP: Interpeduncular nucleus
LG: Lateral geniculate nucleus
M: Mamillary body
MB: Midbrain
MBbp: Midbrain basal plate
Me: Medullary domain (Medulla Oblongata)
mlf: Medial longitudinal fasciculus
mp1: Midbrain prosomere 1
mp2: Midbrain prosomere 2
mtg: Mammillotegmental tract
mth: Mammillothalamic tract
NH: Neurohypophysis
nst/mc: Nigrostriatal/mesolimbic/mesocortical tract
OB: Olfactory bulb
och: Optic chiasm
ot: Optic tract
P: Pons/Pontine domain
PAG: Periaqueductal gray
pc: Posterior commissure
PG: Pineal Gland
PHy: Peduncular hypothalamus
POA: Preoptic area
PrP: Prepontine domain
PT: Pretectum
PTh: Prethalamus
r0: Rhombomere 0
r1r: Rhombomere 1 rostral
r1c: Rhombomere 1 caudal
r2: Rhombomere 2
r3: Rhombomere 3
r4: Rhombomere 4
r5: Rhombomere 5
r6: Rhombomere 6
r7: Rhombomere 7
r8: Rhombomere 8
r9: Rhombomere 9
r10: Rhombomere 10
r11: Rhombomere 11
rf: Retroflex fasciculus (tract)
RP: Retropontine domain
RMC: Red magnocellular nucleus
RPC: Red parvocellular nucleus
Rt: Reticular nucleus
SC: Superior colliculus
Se: Septum
SN: Substance nigra
SNc: Substance nigra, compact
SNp: Substance nigra, primordium
SNr: Substance nigra, reticular
St: Striatum
STh: Subthalamic nucleus
Te: Telencephalon
TG: Tectal gray
Th: Thalamus
THy: Terminal hypothalamus
Tu: Olfactory tubercle
VTA: Ventral tecmental area
VTAp: Ventral tecmental area, primordium
III: Third ventricle
